# Supplementary material for: Characteristic Profile of the Hazardous, Nutritional, and Taste-Contributing Compounds during the Growth of Argopecten irradians with Different Shell Colors
Source: Foods. 2023 Dec 2;12(23):4354. doi: 10.3390/foods12234354 (PMC10706102; doi:10.3390/foods12234354)
Supplement: Supplementary file 1 [file foods-12-04354-s001.zip › foods-2707374-supplementary.pdf]

Methods of analysis

Moisture was determined using the direct drying method [1]. Crude fat was determined by means of the Soxhlet extraction method [2]. Crude protein was determined and analyzed using the Kjeldahl method [3]. Fatty acids were determined using gas chromatography normalization [4]. Amino acids were determined using the amino acid analyzer method [5]. The contents of nine minerals, namely, potassium (K), sodium (Na), calcium (Ca), magnesium (Mg), iron (Fe), copper (Cu), manganese (Mn), zinc (Zn), and phosphorus (P) were determined with the inductively coupled plasma mass spectrometry (ICP-MS) method [6]. The selenium (Se) content was determined using atomic fluorescence spectrometry [7]. Water-soluble vitamins of extract method were optimized [8], and the extraction solvent was instead of 10 mL 0.1 % hydrochloric acid solution, and the extract was finally dissolved in 10 mM ammonium formate solution before injecting into the LCMS. The instrument parameter was re-optimized by the Kinetex XB C18 column (2.1 × 100 mm, 2.6 μm). Fat-soluble vitamin analysis was optimized [9]. Kinetex XB C18 column (2.1 × 100 mm, 2.6 μm) was performed, and the two mobile phase solvents were methanol containing 0.1 % formic acid and 0.1 % formic acid aqueous solution.

The analytical method of free amino acids (FAAs), nucleotides, organic acids, betaine, and trimethylamine oxide were optimized [10-12] as follows: 0.1 g sample (the result in Table S13 is the wet weight content converted according to the moisture content) was mixed with 20 mL of 0.1 M HCl, vortexed for 2 min, then sonicated for 15 min (40 MHz) and heated at 75 ◦C for 10 min. After the sample was cooled to room temperature, it was centrifuged at 6000 r/min for 10 min. Following centrifugation, 40 μL of the clear supernatant was further diluted with 960 μL of MS-grade water and vortexed for 10 s. The mixture was filtered through a 0.22-μm PES syringe filter, and 2 μL of this mixture was injected into the Sciex 5500 Qtrap mass spectrometer system. Chromatographic separation was performed on an intrada amino acid column (3 ×100 mm, 3.0 μm) for analyzing FAAs. Waters ACQUITY UPLC HSS T3 column (2.1 × 100 mm, 1.8 μm) was employed to determine nucleotides, organic acids, betaine, and trimethylamine oxide.

E. coli was measured using the Most Probable Number (MPN) method, a poisson distribution-based indirect count method [13]. *V. parahaemolyticus* was measured using the MPN method [14]. Reference for four elements, namely, lead, cadmium, mercury, and total arsenic in harmful heavy metals were determined in accordance with the ICP-MS method [15]. Polycyclic aromatic hydrocarbons were determined by applying liquid chromatography tandem mass spectrometry [16]. Polychlorinated biphenyls were determined using gas chromatography [17]. Paralytic toxins were determined using LC-MS/MS [18]. Soluble toxins were determined by means of LC-MS/MS [19]. All analyses were performed in triplicate (n = 3).

Reference:

[1] National Standards of the People's Republic of China, 2016. Determination of Moisture in Food GB 5009.3-2016. Health and Family Planning Committee and National Medical Products Administration, People's Republic of China.

[2] National Standards of the People's Republic of China, 2016. National Food Safety Standards-determination of Fat in Food GB 5009.6-2016. Health and Family Planning Committee and National Medical Products Administration, People's Republic of China.

[3] National Standards of the People's Republic of China, 2016. Determination of Protein in Food GB 5009.5-2016. Health and Family Planning Committee and National Medical Products Administration, People's Republic of China.

[4] National Standards of the People's Republic of China, 2016d. National Food Safety Standards-determination of Fatty Acid in Food GB 5009.168-2016. Health and Family Planning Committee and National Medical Products Administration, People's Republic of China.

[5] National Standards of the People's Republic of China, 2016e. Determination of Amino Acids in Food GB 5009.124-2016. Health and Family Planning Committee And National Medical Products Administration, People's Republic of China.

[6] National Standards of the People's Republic of China, 2016f. Determination of Multi elements in Food GB 5009.268-2016. Health and Family Planning Committee and National Medical Products Administration, People's Republic of China.

[7] National Standards of the People's Republic of China, 2007. National Standards for the

Determination of Se in food GB 5009.93-2007. Health and Family Planning Committee and National Medical Products Administration, People's Republic of China.
[8] Lin, D.Z., Diao, J.Y., Lei, C.Y., Yue, M., Sun, Z.H., Zang, Z., 2020. Simultaneous detection of serum levels of 19 vitamins based on LC-MS/MS. J. Guangdong Med. Univ. 38 (4), 400–406.
[9] Yan, H., Cui, F.Y., Bie, W., Feng, X., Liang, N.N., Zhang, Z.H., 2018. Simultaneous determination of 5 kinds of fat-soluble vitamins in infant formula milk powder by stable isotope dilution/liquid chromatography-tandem mass spectrometry. J. Food Saf. Qual. 9 (23), 6130–6139
[10] Zhang, X., Cheng, J., Han, D., Chen, X., Liu, Y., 2019a. Regional differences in fatty acid composition of sea cucumber (*Apostichopus japonicus*) and scallop (*Patinopecten yesoensis*) in the coastal areas of China. Reg. Stud. Mar. Sci. 31, 100782
[11] Zhang, N.L., Ayed, C., Wang, W.L., Liu, Y., 2019b. Sensory-guided analysis of key taste active compounds in pufferfish (Takifugu obscurus). J. Agric. Food Chem. 67 (50), 13809–13816.
[12] Flores, P., Hellín, P., Fenoll, J., 2012. Determination of organic acids in fruits and vegetables by liquid chromatography with tandem-mass spectrometry. Food Chem. 132 (2), 1049–1054.
[13] National Food Safety Standards of China, 2012. Food Microbiology test Escherichia coli Count GB 4789.38-2012. Health and Family Planning Committee and National Medical Products Administration, People's Republic of China.
[14] National Food Safety Standards of China, 2013. Food Microbiology Test Vibrio Parahaemolyticus Test GB 4789.7-2013. Health and Family Planning Committee and National Medical Products Administration, People's Republic of China
[15] National Standards of the People's Republic of China, 2016f. Determination of Multi elements in Food GB 5009.268-2016. Health and Family Planning Committee and National Medical Products Administration, People's Republic of China.
[16] Guo, M.M., Wu, H.Y., Yang, F., Tan, Z.J., Li, Z.X., Zhai, Y.X., 2013. Determination of 16 polycyclic aromatic hydrocarbons in fishery products by improved QuEChERS-high performance liquid chromatography. Environ. Chem. 32 (6), 1025–1031
[17] National Food Safety Standards of China, 2014. Food-Determination of indicative polychlorinated biphenyls GB 5009.190-2014. Health and Family Planning Committee and National Medical Products Administration, People's Republic of China.
[18] National Food Safety Standards of China, 2016g. Determination of Paralytic Shellfish Toxins in Shellfish GB 5009.213-2016. Health and Family Planning Committee and National Medical Products Administration, People's Republic of China.
[19] National Food Safety Standards of China, 2016h. Determination of Diarrhoeal Shellfish Toxin in Shellfish GB 5009.212-2016. Health and Family Planning Committee and National Medical Products Administration, People's Republic of China.

Table S1

Variation of water quality parameters in sampling area

Parameters	August	September	October
Temperature (°C)	21.2±0.9^{a}	23.5±1.0^{a}	17.1±0.9^{b}
Salinity	26.70±0.50^{a}	23.20±0.90^{c}	24.50±0.50^{b}
Dissolved oxygen (mg L^{-1})	6.31±0.15^{c}	7.33±0.11^{b}	9.79±0.13^{a}
pH	7.87±0.08^{b}	7.84±0.09^{b}	8.16±0.05^{a}
Chlorophyll a (μg L^{-1})	3.78±0.03^{a}	2.28±0.02^{b}	1.71±0.01^{c}
NH_4-N (mg L^{-1})	0.02±0.00^{b}	0.10±0.00^{a}	0.12±0.00^{a}
NO_3-N (mg L^{-1})	0.09±0.00^{b}	1.11±0.01^{a}	1.00±0.01^{a}
NO_2-N (mg L^{-1})	0.01±0.00^{b}	0.09±0.00^{a}	0.07±0.00^{a}
PO_4-P (mg L^{-1})	0.02±0.00^{c}	0.10±0.00^{a}	0.07±0.00^{b}
DIN (mg L^{-1})	0.12±0.00^{b}	1.31±0.01^{a}	1.19±0.01^{a}
N/P	5.06±0.05^{b}	13.64±0.09^{a}	17.05±0.11^{a}

Values are presented as means ± SD.

Values in a same column that do not share a same superscript are significantly different ($p < 0.05$).

Table S2

Microbial contents of *A. irradians* during growth (MPN/g)

Bacteria	Golden Shell			Purple Shell		
	August	September	October	August	September	October
Escherichia coli	0.62±0.12	2.10±0.10	2.10±0.08	2.30±0.04	2.60±0.10	1.50±0.05
Vibrio parahaemolyticus	7.20±1.20	11.00±0.10	23.00±0.80	11.00±0.06	12.00±0.08	11.00±0.05

Values are presented as means ± SD. ND: means not detected.

Table S3

Heavy metals contents of *A. irradians* during growth

Compounds	Golden Shell			Purple Shell		
	August	September	October	August	September	October
Pb (mg/kg)	0.12±0.00	0.27±0.01	0.19±0.01	0.10±0.00	0.28±0.01	0.23±0.01
Cd （mg/kg）	0.38±0.02	0.54±0.01	0.49±0.01	0.35±0.01	0.63±0.02	0.66±0.01
As (mg/kg)	0.41±0.04	0.34±0.04	0.35±0.02	0.24±0.02	0.31±0.01	0.35±0.03
Hg (μg/kg)	10.31±0.07	14.36±0.04	16.06±0.04	13.82±0.59	10.37±0.06	14.53±0.66

Values are presented as means ± SD. ND: means not detected.

Table S4

Contents of polychlorinated biphenyls of *A. irradians* during growth (μg/kg)

Compounds	Golden Shell			Purple Shell		
	August	September	October	August	September	October
PCB28	ND	ND	ND	ND	ND	ND
PCB52	0.91±0.01	0.92±0.12	2.97±0.21	1.03±0.01	1.11±0.13	2.77±0.18
PCB101	ND	4.12±0.21	15.18±0.11	0.81±0.04	3.65±0.30	11.62±0.04
PCB118	ND	2.35±0.00	9.57±0.19	ND	2.66±0.10	1.50±0.06
PCB180	ND	1.43±0.08	1.50±0.04	ND	ND	1.19±0.08
PCB138	ND	ND	ND	ND	ND	ND
PCB153	ND	ND	ND	ND	ND	ND
Total	0.91±0.01	8.81±0.07	29.22±0.09	1.84±0.11	8.60±0.04	17.08±0.14

Values are presented as means ± SD. ND: means not detected.

Table S5

Contents of polycyclic aromatic hydrocarbons of *A. irradians* during growth (μg/kg)

Compounds	Golden Shell			Purple Shell		
	August	September	October	August	September	October
Naphthalene	2.44±0.02	ND	ND	ND	ND	ND
Acenaphthene	5.68±0.05	12.25±0.03	5.69±0.03	13.53±0.08	17.06±0.10	11.31±0.07
Fluornene	23.30±0.15	4.17±0.03	2.19±0.01	5.49±0.02	4.87±0.03	6.06±0.03
Phenanthrene	13.00±0.10	48.16±0.15	20.23±0.13	23.16±0.12	32.33±0.21	25.25±0.12
Anthracene	18.44±0.10	24.10±0.12	13.02±0.06	13.10±0.07	18.29±0.08	22.34±0.12
Fluoranthene	12.16±0.04	31.25±0.16	ND	17.40±0.12	37.64±0.22	29.73±0.22
Pyrene	8.45±0.03	7.10±0.02	ND	11.69±0.02	7.05±0.02	ND
Benz [a] anthracene	5.41±0.03	16.73±0.04	13.33±0.05	7.45±0.03	18.12±0.08	12.96±0.09

Chrysene	4.28±0.02	21.98±0.12	3.36±0.01	5.59±0.02	18.93±0.10	3.70±0.01
Benzo [b] fluoranthene	2.44±0.01	35.82±0.12	2.12±0.01	5.39±0.02	36.29±0.21	ND
Benzo [k] fluoranthene	ND	ND	ND	ND	ND	ND
Benzo [a] pyrene	ND	ND	ND	ND	ND	ND
Dibenz [a, h] anthracene	5.32±0.35	ND	ND	ND	ND	ND
Benzo [g, h, i] perylene	ND	ND	ND	ND	ND	ND
Indeno[1,2,3-c,d] pyrene I	ND	ND	ND	ND	ND	ND
Acenaphthylene	ND	ND	ND	ND	ND	ND

Values are presented as means ± SD. ND: means not detected.

Table S6

Contents of paralytic shellfish poison of *A. irradians* during growth (μg/kg)

Compounds	Golden Shell			Purple Shell		
	August	September	October	August	September	October
STX	ND	ND	ND	ND	ND	ND
dcSTX	ND	ND	ND	ND	ND	ND
neoSTX	ND	ND	ND	ND	ND	ND
GTX2	ND	ND	ND	ND	ND	ND
GTX1	ND	ND	ND	ND	ND	ND
GTX5	ND	ND	ND	ND	ND	ND
dcGTX2	ND	ND	ND	ND	ND	ND
GTX3	ND	ND	ND	ND	ND	ND
GTX4	ND	ND	ND	ND	ND	ND
dcGTX3	ND	ND	ND	ND	ND	ND
Total (μgSTX/kg)	ND	ND	ND	ND	ND	ND

Values are presented as means ± SD. ND: means not detected.

Table S7

Contents of fat-soluble shellfish poison of *A. irradians* during growth (μg/kg)

Compounds	Golden Shell			Purple Shell		
	August	September	October	August	September	October
OA	ND	ND	ND	ND	ND	ND
DTX-1	ND	ND	ND	ND	ND	ND
DTX-2	ND	ND	ND	ND	ND	ND
Total(μgOAeq/kg)	ND	ND	ND	ND	ND	ND

Values are presented as means ± SD. ND: means not detected.

Table S8

Changes in Proximate composition of *A. irradians* during growth (g/100 g)

Index	Golden shell			Purple shell		
	August	September	October	August	September	October
Moisture	79.50±0.09^{c}	85.67±0.34^{b}	89.06±0.23^{a}	81.73±0.70^{c}	85.18±0.41^{b}	89.18±0.14^{a}
*Protein	58.05±0.05^{c}	60.80±0.05^{b}	64.39±0.05^{a}	56.60±0.05^{e}	56.10±0.05^{f}	57.41±0.37^{d}
*Fat	9.56±0.23^{b}	7.23±0.58^{c}	4.77±0.23^{d}	11.03±0.47^{a}	5.06±0.10^{d}	4.48±0.22^{d}

Values are presented as means ± SD.
Values in a same column that do not share a same superscript are significantly different ($p < 0.05$).
* indicates that it was measured with dry samples.

Table S9

Hydrolyzed amino acid contents of *A. irradians* during growth (g/100 g)

Amino acids	Golden Shell			Purple Shell		
	August	September	October	August	September	October

Alanine	1.96±0.05^{ab}	2.08±0.10^{a}	1.94±0.09^{ab}	1.88±0.09^{b}	1.90±0.11^{b}	1.88±0.09^{b}
Arginine	5.34±0.22^{bc}	5.62±0.31^{abc}	6.22±0.20^{a}	5.08±0.04^{c}	5.91±0.42^{ab}	6.16±0.49^{a}
Glycine	9.25±0.65^{d}	20.97±0.26^{a}	10.05±0.15^{c}	10.31±0.43^{c}	10.32±0.54^{c}	12.65±0.40^{b}
*Methionine	0.89±0.04^{b}	0.94±0.03^{ab}	1.02±0.08^{a}	0.84±0.01^{b}	0.93±0.09^{ab}	0.94±0.06^{ab}
*Phenylalanine	1.08±0.07^{ab}	0.97±0.08^{c}	1.11±0.03^{a}	1.02±0.04^{abc}	1.00±0.02^{bc}	1.06±0.04^{abc}
Proline	0.86±0.02^{b}	0.93±0.01^{a}	0.93±0.01^{a}	0.82±0.01^{c}	0.88±0.03^{b}	0.86±0.01^{b}
*Threonine	1.90±0.06^{ab}	2.13±0.24^{a}	2.02±0.17^{ab}	1.77±0.13^{b}	1.87±0.05^{ab}	1.86±0.10^{b}
Tyrosine	1.19±0.02^{a}	1.39±0.03^{a}	1.37±0.15^{a}	1.35±0.14^{a}	1.23±0.06^{a}	1.25±0.09^{a}
*Valine	1.26±0.09^{a}	1.23±0.12^{a}	1.30±0.03^{a}	1.21±0.04^{a}	1.25±0.07^{a}	1.23±0.05^{a}
Aspartic Acid	4.81±0.31^{a}	5.06±0.25^{a}	5.05±0.15^{a}	4.37±0.16^{b}	4.73±0.10^{a}	4.99±0.10^{a}
Glutamic Acid	4.62±0.19^{ab}	5.05±0.52^{a}	4.89±0.04^{ab}	4.49±0.25^{b}	4.55±0.23^{ab}	4.76±0.08^{ab}
Histidine	1.10±0.02^{c}	1.37±0.15^{a}	1.31±0.07^{ab}	1.16±0.10^{bc}	1.28±0.06^{ab}	1.32±0.11^{ab}
Serine	1.53±0.09^{a}	1.65±0.09^{a}	1.58±0.05^{a}	1.29±0.10^{b}	1.51±0.08^{a}	1.47±0.14^{a}
*Leucine	2.40±0.10^{bc}	2.64±0.06^{a}	2.54±0.16^{ab}	2.19±0.09^{c}	2.46±0.11^{ab}	2.40±0.15^{bc}
*Isoleucine	1.54±0.14^{a}	1.71±0.01^{a}	1.58±0.18^{a}	1.33±0.07^{b}	1.52±0.07^{ab}	1.53±0.09^{ab}
*Lysine	3.11±0.27^{a}	3.17±0.17^{a}	3.04±0.34^{a}	2.93±0.16^{a}	3.07±0.15^{a}	2.98±0.10^{a}
EAA	12.19±0.61^{ab}	12.81±0.26^{a}	12.62±0.37^{ab}	11.31±0.46^{c}	12.10±0.18^{ab}	12.01±0.26^{b}
TAA	42.90±1.09^{de}	56.99±1.18^{a}	46.02±0.28^{bc}	42.10±1.63^{e}	44.44±0.28^{cd}	47.45±1.29^{b}

Values are presented as means ± SD.
Values in a same column that do not share a same superscript are significantly different ($P < 0.05$).
* indicates that it was essential amino acid.

Table S10

The fatty acid content of *A. irradians* during growth (mg/100g)

Fatty acids	Golden Shell			Purple Shell		
	August	September	October	August	September	October
C14:0	132.42±5.54^{b}	39.77±1.91^{d}	102.82±5.90^{c}	171.01±18.5^{a}	101.24±5.18^{c}	87.12±4.47^{c}
C15:0	20.12±1.93^{bc}	16.83±1.70^{c}	21.50±0.43^{b}	27.36±1.71^{a}	19.81±0.15^{bc}	19.87±1.23^{bc}
C16:0	800.20±57.95^{b}	251.02±13.90^{d}	599.08±18.50^{c}	1044.96±81.27^{a}	530.49±3.96^{c}	535.29±44.75^{c}
C17:0	28.10±3.69^{bc}	15.72±1.12^{d}	32.61±1.54^{b}	41.77±3.34^{a}	20.18±1.43^{d}	26.64±1.63^{c}
C18:0	136.03±10.60^{cd}	77.15±7.25^{e}	144.44±3.57^{bc}	174.97±13.99^{a}	161.65±4.14^{ab}	117.59±7.97^{d}
SFA	1116.87±74.00^{b}	400.49±21.27^{d}	900.45±29.74^{c}	1460.08±118.24^{a}	833.36±4.08^{c}	786.51±56.08^{c}
C16:1n7	271.60±19.98^{a}	46.36±2.27^{c}	69.46±2.18^{bc}	258.63±20.76^{a}	283.54±11.33^{a}	81.18±3.37^{b}
C18:1n9c	84.69±7.59^{b}	43.59±0.81^{d}	73.43±2.82^{b}	112.21±8.67^{a}	58.91±1.86^{c}	53.64±4.51^{cd}
C20:1	462.16±29.61^{b}	177.35±10.16^{e}	396.10±10.42^{c}	535.62±45.12^{a}	54.70±0.03^{f}	330.41±21.61^{d}
MUFA	818.45±56.53^{a}	267.30±12.86^{d}	538.99±15.37^{b}	906.46±74.43^{a}	397.14±13.16^{c}	465.23±28.63^{bc}
C18:2n6c (LA)	69.39±4.50^{a}	22.77±3.29^{c}	56.34±1.25^{b}	77.70±6.48^{a}	50.38±2.12^{b}	49.76±3.11^{b}

C18:3n3 (LNA)	9.75±1.36^{c}	20.70±2.09^{b}	19.28±1.87^{b}	8.28±0.47^{c}	47.01±0.70^{a}	11.39±0.23^{c}
C20:2	27.85±1.90^{a}	9.96±0.45^{c}	18.79±0.53^{b}	19.14±0.94^{b}	19.74±1.71^{b}	26.30±2.58^{a}
C20:4n6 (ARA)	46.56±2.07^{c}	42.48±3.96^{c}	84.18±3.73^{a}	61.45±7.28^{b}	70.7±3.21^{b}	61.97±2.98^{b}
C20:5 (EPA)	985.43±72.10^{b}	439.00±35.96^{d}	873.43±62.35^{bc}	1129.02±97.32^{a}	487.00±4.11^{d}	763.55±48.75^{c}
C22:6n3 (DHA)	659.60±43.38^{a}	318.02±16.37^{c}	654.01±14.53^{a}	740.46±68.23^{a}	479.80±16.57^{b}	522.78±27.90^{b}
PUFA	1798.57±121.49^{b}	852.94±28.25^{e}	1706.03±74.69^{b}	2036.05±177.75^{a}	1154.64±16.79^{d}	1435.76±82.70^{c}
PUFAn-3	1654.77±114.53^{b}	777.72±33.54^{e}	1546.73±70.08^{b}	1877.76±165.53^{a}	1013.81±13.17^{d}	1297.72±76.69^{c}
PUFAn-6	115.95±6.54^{b}	65.25±7.04^{c}	140.52±4.67^{a}	139.15±13.13^{a}	121.08±5.32^{b}	111.73±6.07^{b}
n-3/n-6	14.27±0.31^{a}	12.04±1.71^{bc}	11.01±0.23^{c}	13.50±0.40^{ab}	8.38±0.27^{d}	11.61±0.06^{c}
AI	0.51±0.01^{b}	0.37±0.01^{d}	0.45±0.02^{c}	0.59±0.01^{a}	0.60±0.01^{a}	0.47±0.01^{c}
TI	0.20±0.01^{b}	0.15±0.01^{d}	0.17±0.01^{c}	0.23±0.01^{a}	0.24±0.01^{a}	0.18±0.01^{c}
EFA	79.14±3.97^{bc}	43.47±3.32^{e}	75.62±3.11^{c}	85.98±6.47^{b}	97.39±2.82^{a}	61.15±3.33^{d}
HH	1.99±0.02^{c}	3.05±0.22^{a}	2.51±0.11^{b}	1.75±0.03^{c}	1.89±0.02^{c}	2.35±0.04^{b}
SFA/PUFA	0.62±0.01^{b}	0.47±0.04^{d}	0.53±0.02^{c}	0.72±0.01^{a}	0.72±0.01^{a}	0.55±0.01^{c}

Values are presented as means ± SD.
Values in a same column that do not share a same superscript are significantly different ($p < 0.05$).

Table S11

The fatty acid composition of *A. irradians* during growth (% total fatty acid)

Fatty acids	Golden Shell			Purple Shell		
	August	September	October	August	September	October
C14:0	3.55±0.13^{c}	2.62±0.14^{d}	3.27±0.13^{c}	3.88±0.15^{b}	4.24±0.20^{a}	3.25±0.24^{c}
C15:0	0.54±0.05^{e}	1.11±0.11^{a}	0.68±0.01^{cd}	0.62±0.01^{de}	0.83±0.01^{b}	0.74±0.01^{c}
C16:0	21.43±0.14^{c}	16.50±0.85^{f}	19.05±0.47^{e}	23.74±0.18^{a}	22.24±0.24^{b}	19.9±0.43^{d}
C17:0	0.76±0.11^{b}	1.03±0.07^{a}	1.04±0.04^{a}	0.95±0.01^{ab}	0.85±0.06^{b}	0.99±0.01^{a}
C18:0	3.64±0.04^{d}	5.07±0.46^{b}	4.59±0.07^{c}	3.98±0.02^{d}	6.78±0.15^{a}	4.37±0.03^{c}
SFA	29.91±0.04^{c}	26.33±1.30^{e}	28.63±0.70^{d}	33.17±0.25^{b}	34.94±0.06^{a}	29.25±0.30^{cd}
C16:1n7	7.27±0.08^{b}	3.05±0.14^{d}	2.21±0.04^{e}	5.88±0.04^{c}	11.89±0.51^{a}	3.02±0.13^{d}
C18:1n9c	2.27±0.08^{c}	2.87±0.06^{a}	2.33±0.05^{c}	2.55±0.04^{b}	2.47±0.09^{b}	1.99±0.04^{d}
C20:1	12.38±0.06^{a}	11.66±0.64^{b}	12.60±0.18^{a}	12.17±0.01^{a}	2.29±0.01^{c}	12.29±0.06^{a}
MUFA	21.92±0.05^{a}	17.58±0.81^{c}	17.14±0.26^{cd}	20.59±0.06^{b}	16.65±0.61^{d}	17.31±0.12^{cd}
C18:2n6c (LA)	1.86±0.02^{b}	1.50±0.21^{c}	1.79±0.04^{b}	1.76±0.02^{b}	2.11±0.08^{a}	1.85±0.01^{b}
C18:3n3 (LNA)	0.26±0.05^{e}	1.36±0.14^{b}	0.61±0.05^{c}	0.19±0.02^{e}	1.97±0.02^{a}	0.42±0.02^{d}
C20:2	0.75±0.06^{bc}	0.66±0.03^{cd}	0.60±0.02^{d}	0.44±0.05^{e}	0.83±0.07^{b}	0.98±0.12^{a}
C20:4n6 (ARA)	1.25±0.04^{d}	2.79±0.25^{ab}	2.68±0.03^{b}	1.39±0.09^{d}	2.96±0.13^{a}	2.31±0.04^{c}
C20:5 (EPA)	26.38±0.18^{bc}	28.87±2.41^{a}	27.75±1.33^{ab}	25.64±0.22^{c}	20.42±0.24^{d}	28.41±0.05^{ab}

C22:6n3 (DHA)	17.67±0.08^{c}	20.91±1.14^{a}	20.80±0.38^{a}	16.81±0.18^{c}	20.11±0.63^{ab}	19.46±0.23^{b}
PUFA	48.17±0.06^{c}	56.09±2.02^{a}	54.23±0.96^{b}	46.24±0.32^{d}	48.41±0.55^{c}	53.43±0.35^{b}

Values are presented as means ± SD.
Values in a same column that do not share a same superscript are significantly different (*p* < 0.05).

Table S12

Vitamin contents of *A. irradians* during growth

Vitamins	Golden Shell			Purple Shell		
	August	September	October	August	September	October
VB_1 (μg/100 g)	39.83±1.16^{a}	21.59±1.50^{c}	22.09±2.74^{c}	22.42±0.62^{c}	26.40±0.42^{b}	19.65±0.09^{c}
VB_2 (μg/100 g)	482.85±0.35^{b}	490.64±9.19^{b}	101.05±1.11^{e}	718.15±21.29^{a}	431.05±16.69^{c}	168.36±2.18^{d}
VB_5 (μg/100 g)	744.07±12.06^{a}	236.54±8.29^{d}	458.55±50.88^{c}	654.33±23.32^{b}	139.25±5.16^{e}	451.65±0.07^{c}
VB_6 (μg/100 g)	34.98±0.08^{a}	34.79±0.30^{a}	36.21±0.16^{a}	35.88±1.51^{a}	34.47±0.81^{a}	34.39±0.24^{a}
VB_{12} (μg /100 g)	5.94±0.05^{b}	7.55±0.41^{a}	4.56±0.58^{c}	6.94±0.05^{a}	7.07±0.45^{a}	5.54±0.51^{bc}
VB_3 (mg/100 g)	2.83±0.02^{a}	1.21±0.12^{d}	0.62±0.01^{f}	2.49±0.09^{b}	1.37±0.02^{c}	1.05±0.04^{e}
VA (μg/100 g)	124.87±12.42^{a}	75.34±2.30^{c}	104.98±3.15^{ab}	105.72±15.19^{ab}	77.88±2.72^{c}	89.32±5.02^{bc}
VE (mg/100 g)	1.24±0.04^{bc}	1.06±0.18^{cd}	3.05±0.01^{a}	0.93±0.06^{d}	0.94±0.03^{d}	1.38±0.14^{b}
VD_2 (μg/100 g)	3.39±0.26^{de}	4.23±0.25^{d}	17.68±0.55^{a}	2.52±0.12^{e}	7.80±0.98^{c}	16.08±0.65^{b}
VD_3 (μg/100 g)	5.22±0.38^{abc}	5.81±0.39^{abc}	6.55±0.20^{a}	4.51±0.09^{c}	5.05±0.43^{bc}	6.23±1.07^{ab}

Values are presented as means ± SD.
Values in a same column that do not share a same superscript are significantly different (*p* < 0.05)

Table S13

Mineral concentrations of *A. irradias* during growth

Elements	Golden Shell			Purple Shell		
	August	September	October	August	September	October
Ca (mg/g)	5.36±0.29^{e}	8.58±0.025^{a}	7.87±0.04^{b}	6.47±0.21^{de}	7.43±0.20^{de}	7.55±0.17^{c}
P (mg/g)	8.75±0.28^{b}	5.47±0.30^{d}	7.24±0.20^{c}	8.01±0.32^{bc}	14.34±0.22^{a}	7.36±0.22^{c}
K (mg/g)	14.43±0.04^{b}	13.73±0.64^{bc}	14.42±0.46^{b}	14.71±0.46^{b}	17.03±0.10^{a}	13.09±0.03^{c}
Mg (mg/g)	1.01±0.01^{b}	1.87±0.10^{a}	0.91±0.04^{bc}	0.86±0.02^{d}	1.07±0.02^{b}	0.82±0.01^{c}
Fe (mg/kg)	152.90±9.75^{d}	377.13±5.93^{a}	174.93±5.338^{c}	111.63±1.03^{e}	210.00±15.40^{b}	91.02±1.95^{f}
Zn (mg/kg)	621.50±3.54^{d}	904.75±59.75^{b}	1298.75±28.64^{a}	743.25±33.59^{c}	534.00±11.31^{e}	844.50±15.56^{b}
Se (mg/kg)	2.25±0.00^{b}	1.94±0.00^{c}	5.63±0.00^{a}	6.74±0.03^{a}	4.21±0.01^{b}	4.21±0.02^{b}
Cu (mg/kg)	53.20±0.57^{b}	71.40±3.80^{a}	56.51±0.00^{b}	58.28±1.94^{b}	54.61±0.10^{b}	38.03±1.07^{c}
Mn (mg/kg)	23.85±0.35^{c}	32.35±0.76^{a}	14.39±0.31^{e}	17.13±0.19^{d}	26.20±0.86^{b}	14.93±1.38^{e}

Values are presented as means ± SD.
Values in a same column that do not share a same superscript are significantly different (*p* < 0.05).

Table S14

Flavoring substances of concentrations of *A. irradians* during growth (mg/100 g) (ww)

Flavoring substances	Golden Shell			Purple Shell		
	August	September	October	August	September	October
Alanine	116.19±1.91^{a}	49.52±1.78^{d}	51.42±0.80^{d}	110.32±2.02^{b}	71.84±1.88^{c}	51.43±1.37^{d}
Arginine	26.51±0.41^{b}	10.33±0.39^{d}	10.46±0.3^{d}	24.22±0.21^{c}	31.40±0.26^{a}	10.42±0.29^{d}
Glycine	72.10±0.37^{b}	50.04±2.37^{c}	46.37±1.96^{c}	83.33±1.03^{a}	40.39±3.86^{d}	39.87±4.35^{d}
Methionine	17.41±1.05^{a}	4.66±0.15^{e}	6.95±0.34^{d}	10.96±0.36^{b}	8.47±0.32^{c}	9.06±0.12^{c}
Phenylalanine	10.05±0.30^{b}	4.83±0.14^{d}	4.11±0.04^{e}	10.97±0.30^{a}	10.42±0.12^{b}	6.12±0.38^{c}
Proline	135.86±2.13^{a}	38.82±2.58^{e}	58.13±3.58^{d}	130.83±2.26^{b}	18.57±2.38^{f}	73.52±1.83^{c}
Threonine	29.29±0.17^{a}	6.34±0.17^{e}	9.27±0.21^{d}	29.35±0.08^{a}	14.54±0.52^{b}	12.23±0.11^{c}
Tryptophan	3.33±0.20^{a}	1.49±0.06^{d}	1.78±0.02^{c}	3.43±0.16^{a}	2.70±0.01^{b}	3.35±0.04^{a}
Tyrosine	15.60±0.36^{b}	7.62±0.35^{e}	6.47±0.15^{f}	16.49±0.50^{a}	10.48±0.10^{c}	8.21±0.11^{d}
Valine	10.66±0.11^{b}	4.63±0.17^{d}	3.99±0.19^{e}	11.20±0.28^{a}	10.48±0.28^{b}	5.74±0.12^{c}
Aspartic Acid	130.62±1.60^{a}	55.33±2.65^{c}	45.13±2.37^{d}	98.82±2.58^{b}	24.29±0.53^{e}	44.75±2.17^{d}
Glutamic Acid	236.71±4.91^{a}	73.73±3.20^{d}	101.84±5.96^{b}	240.17±2.14^{a}	105.69±0.63^{b}	94.19±3.05^{c}
Histidine	37.52±0.42^{a}	3.59±0.34^{f}	12.39±0.50^{d}	34.20±0.62^{b}	4.92±0.23^{e}	17.02±0.34^{c}
Serine	45.63±0.32^{b}	13.52±0.52^{d}	16.05±0.48^{d}	48.91±3.63^{a}	20.86±0.04^{c}	23.26±1.11^{c}
Leucine	13.88±0.12^{c}	16.75±0.66^{a}	7.15±0.40^{f}	14.86±0.38^{b}	9.38±0.36^{d}	8.57±0.13^{e}

Isoleucine	10.37±0.28[c]	5.35±0.07[e]	4.61±0.11[f]	11.92±0.34[a]	11.50±0.12[b]	6.37±0.21[d]
Lysine	12.44±0.93[b]	5.84±0.49[d]	3.94±0.01[e]	13.69±0.23[a]	10.07±0.14[c]	6.28±0.17[d]
Cysteine	6.99±0.23[a]	3.87±0.07[c]	3.12±0.06[e]	6.35±0.23[b]	3.78±0.07[cd]	3.60±0.04[d]
Taurine	136.41±2.42[b]	150.02±3.44[a]	76.69±2.80[e]	128.77±2.47[bc]	121.30±7.65[c]	92.07±6.16[d]
TFAA	1132.86±6.17[a]	385.62±5.87[f]	450.05±9.47[e]	1068.44±6.42[b]	831.62±7.35[c]	501.63±13.55[d]
XMP	0.43±0.02[a]	0.31±0.02[c]	0.23±0.01[d]	0.39±0.02[b]	0.32±0.02[c]	0.23±0.01[d]
IMP	19.69±0.43[a]	6.14±0.20[d]	3.86±0.19[f]	17.25±0.58[b]	11.91±0.31[c]	5.60±0.11[e]
AMP	11.63±0.20[b]	8.54±0.25[d]	5.70±0.20[e]	11.07±0.71[bc]	18.17±1.44[a]	10.55±0.63[c]
CMP	0.19±0.01[b]	0.13±0.01[d]	0.10±0.01[e]	0.20±0.01[a]	0.17±0.01[c]	0.10±0.01[e]
GMP	1.70±0.04[a]	1.14±0.02[c]	1.11±0.02[c]	1.28±0.05[b]	1.14±0.02[c]	1.66±0.05[a]
UMP	1.17±0.01[c]	1.40±0.04[b]	1.08±0.03[d]	0.95±0.03[e]	1.05±0.03[d]	1.76±0.05[a]
Succinic	73.68±8.46[a]	33.72±0.63[c]	48.79±6.66[b]	48.14±2.49[b]	14.90±0.72[d]	48.76±9.83[b]
Malic	21.11±1.91[a]	11.78±0.55[d]	10.38±1.61[c]	19.16±1.47[b]	5.97±0.52[d]	12.86±2.22[c]
Citric	25.10±2.82[a]	19.98±0.69[c]	23.74±4.33[b]	17.75±0.52[b]	1.96±0.11[c]	20.38±3.54[b]
Tartaric	0.79±0.15[a]	0.63±0.05[ab]	0.53±0.13[b]	0.72±0.03[ab]	0.76±0.08[ab]	0.50±0.07[c]
Betaine	1266.48±155.24[a]	439.45±24.98[d]	448.00±76.60[d]	571.06±16.31[c]	399.18±35.95[d]	722.08±52.32[b]

Values are presented as means ± SD.
Values in a same column that do not share a same superscript are significantly different ($p < 0.05$).
